# An Honest Joker reveals stereotypical beliefs about the face of deception

**DOI:** 10.1038/s41598-023-43716-4

**Published:** 2023-10-03

**Authors:** Xingchen Zhou, Rob Jenkins, Lei Zhu

**Affiliations:** 1 Department of Psychology, Fudan University, Handan Road 220, Shanghai, 200433 SH People’s Republic of China; 2 Department of Psychology, University of York, York, UK

**Keywords:** Human behaviour, Psychophysics

## Abstract

Research on deception detection has mainly focused on *Simple Deception*, in which false information is presented as true. Relatively few studies have examined *Sophisticated Deception*, in which true information is presented as false. Because Sophisticated Deception incentivizes the appearance of dishonesty, it provides a window onto stereotypical beliefs about cues to deception. Here, we adapted the popular Joker Game to elicit spontaneous facial expressions under *Simple Deception*, *Sophisticated Deception*, and *Plain Truth* conditions, comparing facial behaviors in static, dynamic nonspeaking, and dynamic speaking presentations. Facial behaviors were analysed via machine learning using the Facial Action Coding System. Facial activations were more intense and longer lasting in the *Sophisticated Deception* condition than in the *Simple Deception* and *Plain Truth* conditions. More facial action units intensified in the static condition than in the dynamic speaking condition. *Simple Deception* involved leaked facial behaviors of which deceivers were unaware. In contrast, *Sophisticated Deception* involved deliberately leaked facial cues, including stereotypical cues to lying (e.g., gaze aversion). These stereotypes were inaccurate in the sense that they diverged from cues in the *Simple Deception* condition—the actual appearance of deception in this task. Our findings show that different modes of deception can be distinguished via facial action analysis. They also show that stereotypical beliefs concerning cues to deception can inform behavior. To facilitate future research on these topics, the multimodal stimuli developed in this study are available free for scientific use.

## Introduction

Deception is often understood as a deliberate attempt to mislead by presenting falsehood as truth. Although *Simple Deception* of this type is common in everyday life^1,2^, it is not the only type of deception. Another type, known as *Sophisticated Deception*^3^ or second-order deception^4,5^ can arise in competitive situations including political rivalry, warfare, sports, gambling (e.g., poker), business, and diplomacy^5^. *Sophisticated Deception* refers to the seemingly paradoxical strategy of deception through telling the truth^3^.

The distinction between *Simple Deception* and *Sophisticated Deception* is key to the current study. Table 1 compares their features.

**Table 1:** Classification of Plain Truth, Simple Deception and Sophisticated Deception.

Are they presenting the truth?	Do they intend to deceive?	Do they expect to be believed?	Classification
Yes	No	Yes	Plain Truth
No	Yes	Yes	Simple Deception
Yes	Yes	No	Sophisticated Deception

In *Simple Deception*, a deceiver sends false information that is intended and expected to be perceived as true^6^. In *Sophisticated Deception*, the deceiver considers that the recipient expects the deceiver to lie, and that the deceiver’s information will be interpreted as false. In anticipation, the deceiver employs a ‘double bluff’, conveying literal truth that is intended and expected to be perceived as a lie, so that the receiver will believe the opposite^3^. The identification of the *Sophisticated Deception* strategy led to a surge of research interest on its decision-making characteristics, ERP correlates, and neural basis^3–5,7^. However, despite the substantial literature on facial cues to deception^8,9^, no previous study has examined facial cues to *Sophisticated Deception*. This omission is potentially important. If different types of deception have distinct facial markers, conflating them could lead to incorrect analyses and conclusions. To address this issue, the present study compared facial behaviors in *Plain Truth, Simple Deception* and *Sophisticated Deception*. Our study design prioritizes three principles.

First, our main purpose is to determine the facial features of *Sophisticated Deception* in comparison with *Simple Deception*. According to the most influential meta-analysis of deception cues^8^, the most compelling diagnostic facial cues to *Simple Deception* are pupil dilation, chin raising, lip pressing and facial pleasantness. Even for these cues, there is little consensus as to their reliability^10,11^ and the associated effect sizes are typically small. One possible reason might be that the cues are not unique to people using the *Simple Deception* strategy, but also to people who are telling the truth: both try to avoid being suspected. Conversely, those who use the *Sophisticated Deception* strategy seek to make people doubt them through their facial behaviors. In *Sophisticated Deception*, these facial behaviours presumably follow the speaker’s beliefs about facial cues to *Simple Deception*. Those beliefs might not be accurate. Furthermore, lying may impose greater cognitive load than telling the truth^11^. Dynamic cues become relevant here. For example, people may pause more when cognitive demands are high^12^. False smiles last longer than genuine smiles, and the overall duration appears to be different for spontaneous expressions versus deliberate expressions^13–15^. If deception imposes higher cognitive load than truth-telling, and *Sophisticated Deception* imposes higher cognitive load than *Simple Deception,* then dynamic information could facilitate deception detection.

The second principle concerns facial behaviors across different modalities. Everyday emotional expressions often combine facial and vocal components. In some situations, facial expression can convey rich emotional information without accompanying vocal expression. But the ability of different media to facilitate understanding varies^16^. For example, in studies of *Media Richness Theory*^17^, individuals who were looking for deception preferred rich media (e.g., face-to-face communication) over lean media (e.g., telephonic communication). Previous studies have also found that hearing speakers in addition to seeing them increased deception detection accuracy^6^. To date however, no study has examined how speech might influence facial behavior during deception, and specifically, whether visible behavior might be exaggerated (in compensation) when audible behavior is restricted. To test this possibility, we captured dynamic facial behaviors both with and without speaking in addition to static expression.

The third principle concerns the metacognitive level of facial expression under different deception strategies^18^. Expression and perception are two distinct but inter-related processes in nonverbal communication^19,20^. So too in facial deception. As such, the internal information that the deceiver would like to express, the observable facial behavior they actually produce, and the emotional information that the observer infers from the deceiver’s face can diverge. Importantly, deception itself is not easy because the deceiver must hold in mind two mental states—their own and that of the receiver^21^. Deception by telling the truth (*Sophisticated Deception*) adds further difficulty, because it requires a more layered representation of the receiver’s mental state. To separate these cognitive and metacognitive levels, we compared for each deception condition (i) objective facial behaviors, (ii) subjective facial behaviors estimated by the speakers themselves, and (iii) their estimations of the observers’ performance in spotting deceit.

To generate deception samples, we adapted the well-known Joker Game, recording people’s facial behaviors in three deception conditions (*Plain Truth*, *Simple Deception*, and *Sophisticated Deception*) crossed with three dynamism conditions (*Static*, *Dynamic Speaking*, and *Dynamic Nonspeaking*). Based on the preceding literature, our main hypothesis was that more facial indicators would intensify in *Sophisticated Deception* than in *Simple Deception*, especially cues that have previously been implicated in deception—gaze aversion, pupil dilation, chin raised, lip pressing and facial pleasantness—as these may inform stereotypical beliefs about the face of deception. Second, regarding facial expressions in different dynamism states, we hypothesized that the intensity of indicators would increase from rich media (dynamic speaking condition) to lean media (static condition). We expected that, as the richness of media decreases, deceivers must work harder to express their intentions through facial behavior. Third, in terms of metacognitive insight, we hypothesized that deceivers may underestimate or even misjudge their facial behavioral differences under the three strategies, relative to objective measures extracted by machine learning. To obtain objective measures, we coded facial behaviors using OpenFace 2.0^22^—one of the state-of-the-art approaches for extracting facial features in deception detection studies^23–26^. Combining this range of presentation formats with machine learning techniques not only allowed us to verify previous studies that explored single facial states, it also allowed us to compare deceivers’ expressions in different states.

## Methods

### Participants

Sample size was chosen to align with previous studies on deception detection using FACS (i.e. 20–50 participants^27–29^), as well as the results of power analysis provided by G*Power 3^30^. Twenty-two participants were required to achieve an actual power of 0.95 to find an effect of 0.25 and an alpha of 0.05. Forty Chinese students (20 females, 20 males; mean age 19 years; age range 18–23 years) at Fudan University played the role of deceiver in the Joker game in exchange for a small payment. All participants were right-handed with normal vision and without makeup. Participants were also asked to make sure their eyebrows were fully visible. Those who wore nose rings, labrets, or earrings were asked to remove them for the experiment. Participants with experience portraying emotions they do not feel (due to acting experience, e.g., members of drama club) were excluded before the experiment. Written informed consent to participation and to publish identifying images in an online open access publication was obtained from all participants. Ethical approval for the study was approved by the Human Research Ethics Committee of the School of Social Development and Public Policy of Fudan University in accordance with the Declaration of Helsinki.

### Materials and apparatus

A digital video camera (ORHRO, HDR-AC7, Boya Times Technology Corporation, Shenzhen, China) was used to record participants’ facial expressions. Facial behaviors were coded using OpenFace 2.0^22^. A deck of playing cards was used as a prop to help participants understand the game rules and to support their acting.

### Design

The experiment used a within-subject design. Each player attempted each of the three strategies (*Plain Truth*, *Simple Deception*, *Sophisticated Deception*) in a random order, and completed a questionnaire to assess the intensity of their facial movements after each turn. The dependent variables were, i) the objective facial action units intensity, analyzed by machine learning, ii) the subjective facial action units intensity, estimated by players themselves, iii) ratios in Euclidean distance between the selected facial landmarks and iv) the radians of eye gaze direction in world coordinates, averaged for both eyes.

### Procedure

The experimenter first made sure that the participant was familiar with the rules of the standard Joker game, using a deck of playing cards to demonstrate. The experimenter then explained the specifics of the experimental task, including the three deception strategies. In the standard Joker game, players take turns selecting unseen cards from each other’s hands in a bid to form matching pairs that they can discard. Somewhere amongst the players’ cards is a solitary Joker. As this is the only Joker in the game, it can not be discarded as part of a pair. That constraint sets the whole game in motion, as whoever is left holding the Joker at the end of the game loses. To introduce an element of bluffing, the player who selects the card may ask whether the chosen card is ‘safe’ to take. Critically, the two players’ incentives are unaligned here: the selector prefers not to take the Joker; the holder prefers the selector to take the Joker. Our experimental task cuts to this endgame. The participant (in the role of the holder) has only two cards left, one of which is the Joker. The participant’s goal is to influence the selector’s choice via their own facial behavior. The participant may follow a *Plain Truth*, *Simple Deception*, or *Sophisticated Deception* strategy in pursuit of this goal, depending on the cards and the selection. (see Supplementary Information for verbatim task instructions).

Table 2 shows a detailed comparison of each strategy. After making sure participants understood the game and the three strategies, they were asked to sit one meter away from a digital video camera with their backs against the white wall, thinking about their possible facial expressions in the case of each deceptive strategy. During the facial expression capture session under each strategy (*Plain Truth*, *Simple Deception*, *Sophisticated Deception*), participants were asked to face the camera and were recorded in three dynamism conditions: *Static*, *Dynamic Speaking* and *Dynamic Non-speaking*. To make a fair within-subject comparison, we only recorded their facial expressions when their response to the card in each condition was “safe”. This also corresponds to how people react in real life when the opponents ask if the card is safe. The spoken line was fixed as “safe”, but participants were allowed to speak in any tone and any intonation, as long as it fit what they would like to express. The order in which the dynamism strategies were recorded was up to them. All images and video clips were recorded in a quiet environment, with blackout curtains installed for light control. Participants were allowed to use the two poker cards in hands to support their acting. The Joker Game relies on the players’ delight of success in cheating others (intrinsic motivation). To ensure that participants had a strong desire for self-expression when deceiving, we also provided extra cash prizes for deceivers if the observers failed to spot deceits (extrinsic motivation^31^). Participants were told that their acting would be shown to 100 selectors who did not know them before the experiment. After that, each selector would decide whether to choose the card they were told was safe. One trial would be drawn at random, and we would pay out according to the selector’s choice in the selected trial. If the trial was a deception trial and the sender’s facial expression successfully misled the selector to choose the Joker card, they would be paid a cash bonus.

**Table 2:**
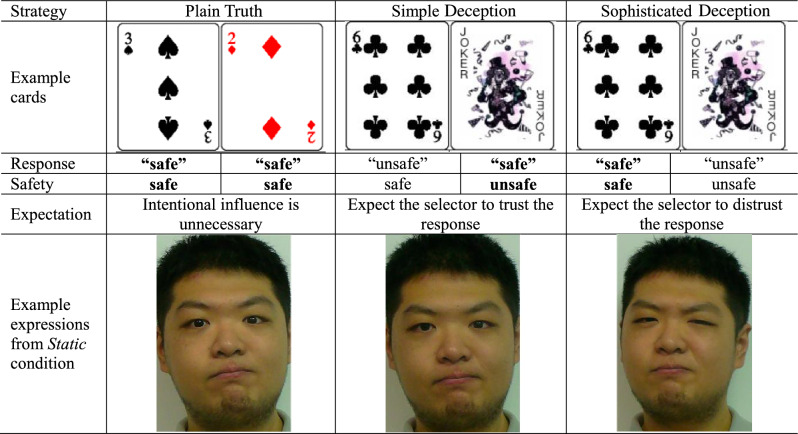
Strategies in the Joker game.

The selector asks the holder whether the selected card is ‘safe’.

Bold refers to actual card condition.

After performing under each strategy, participants completed a retrospective online questionnaire on a tablet concerning, i) the degree that they had engaged with the Joker Game (desire to win; 0–7), ii) their perceived AU intensity (0–5) in the scene just filmed (Whenever the meaning of an AU required clarification, the experimenter described the AU’s location and action direction verbally but did not demonstrate it), and iii) their perceived proportion of selectors who will choose the card they expected (0–100). The entire test session took approximately 50 min to complete. An example of static facial expressions using each strategy from one participant is shown in Table 2.

## Results

**Figure 1:**
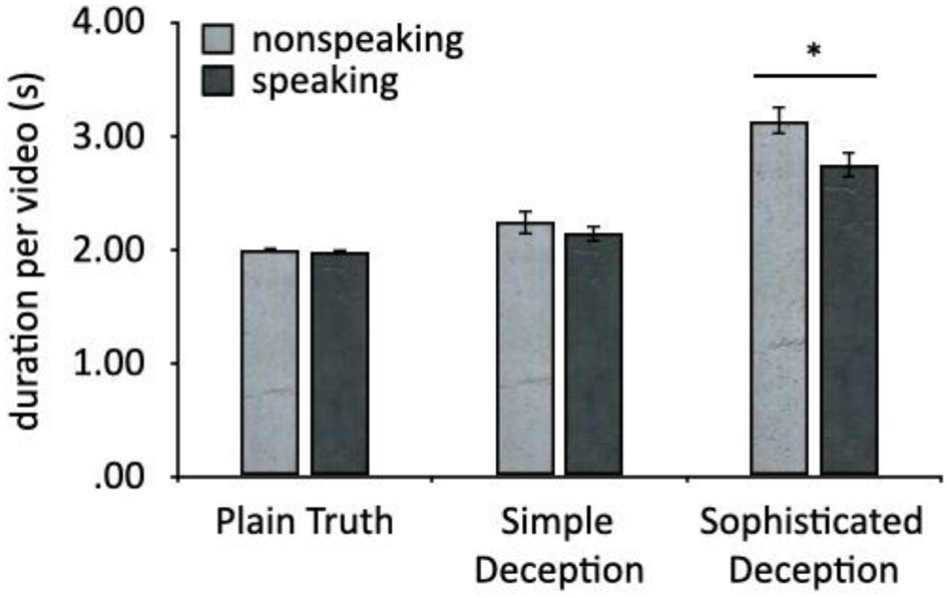
Duration per video split by *Deception* strategy (*Plain Truth*, *Simple Deception*, *Sophisticated Deception*) and *Dynamism* condition (*nonspeaking* and *speaking*). Error bars show SE. Asterisk indicates* p* < .05.

Recording the facial expressions of 40 participants under three deception strategies (*Plain Truth*, *Simple Deception*, *Sophisticated Deception*) with three dynamism conditions for each strategy resulted in 120 images (dimensions: 5600 × 4200; format: JPG) and 569.96 s (434039 frames) in total from 240 videos (dimensions: 1920 × 1080; format: MP4), see Fig. 1 for mean durations in each condition). Participants’ self-reported level of engagement in the competitive game (desire to win) was 6 out of 7 on average, significantly higher than the middle score (3.5), *t* (119) = 27.23, *p* < 0.001, *d* = 2.49. These high scores indicate that participants had correctly understood the game rules and that our recordings in each condition captured genuine reactions.

To determine whether facial behaviors were significantly different across the three deception strategies, we used machine learning software OpenFace 2.0^22^ for facial behavior detection and recognition. Three kinds of indicators were available for the current study, i) the intensity scores (range: 0–5, with 5 indicating the highest intensity) for 17 facial expression action units (AU1, AU2, AU4, AU5, AU6, AU7, AU9, AU10, AU12, AU14, AU15, AU17, AU20, AU23, AU25, AU26 and AU45); ii) the radians of eye gaze direction in world coordinates, averaged across both eyes; iii) the locations of 68 face landmarks and 56 eye region landmarks. The straight-line distance between any two landmarks can be calculated according to the Euclidean distance equation*.* Following Beh and Goh^32^, we selected six representative indicators from these 2D landmarks at the eyebrow, mouth, and eye regions, including the ratio of each eyebrow, the ratio of mouth, the diameter of each pupil and the mean distance between upper and lower eyelids for each eye. All equations and the landmark index followed Beh and Goh^32^ and were integrated through Excel; see supplementary information for details.

In comparing performance across deception strategies, we analyzed data at both cognitive and metacognitive levels. The cognitive analysis considered both indirect analysis (duration of acting in videos) and direct analysis of 25 indicators (17 objective action units’ intensity, 6 landmark distance indicators, and radians in the horizontal and vertical eye gaze direction). The metacognitive analysis included participants’ estimations of themselves (21 subjective action units’ intensity) and that of others (the proportion of selectors who will choose the card they expected).

### Cognitive analysis

#### Indirect analysis

##### Duration of acting in videos

Since participants were asked to act spontaneously for any length of time in the three situations, the duration of their acting in the two dynamic conditions varied (see Fig. 1). To test the prediction that deceptive acts would last longer than truthful acts, duration data were submitted to a 2 × 3 repeated-measure ANOVA with the within-subjects factors of *Dynamism* (*dynamic without speaking*, *dynamic speaking*) and *Deception Strategy* (*Plain Truth*, *Simple Deception*, *Sophisticated Deception*). The main effect of *Dynamism* was significant, with longer duration in the nonspeaking condition (*M* = 2.46, *SE* = 0.06) than that in the speaking condition (*M* = 2.29, *SE* = 0.05) [*F* (1, 39) = 4.86, *p* = 0.03, η_p_^2^ = 0.11]. The main effect of *Deception Strategy* was also significant, with duration increasing from the *Plain Truth* condition (*M* = 1.99, *SE* = 0.01) through the *Simple Deception* condition (*M* = 2.19, *SE* = 0.08) to the *Sophisticated Deception* condition (*M* = 2.94, *SE* = 0.09) [*F* (2, 78) = 54.62, *p* < 0.001, η_p_^2^ = 0.58]. These main effects were qualified by a significant interaction between *Dynamism* and *Deception Strategy* [*F* (2, 78) = 3.89, *p* = 0.02, η_p_^2^ = 0.09]. Simple main effects confirmed that, in the *Sophisticated Deception* condition, the duration of nonspeaking clips exceeded the duration of speaking clips [*F* (1, 39) = 6.50, *p* = 0.02, η_p_^2^ = 0.14]. This difference did not emerge in the *Plain Truth* condition [*F* (1, 39) = 0.28, *p* = 0.60, η_p_^2^ = 0.01] or the *Simple Deception* condition [*F* (1, 39) = 0.69, *p* = 0.41, η_p_^2^ = 0.02]. The simple main effect of *Deception Strategy* was significant for both nonspeaking clips [*F* (2,38) = 40.54, *p* < 0.001, η_p_^2^ = 0.68] and speaking clips [*F* (2, 38) = 20.75, *p* < 0.001, η_p_^2^ = 0.52]. In both *Dynamism* conditions (nonspeaking and speaking), *Sophisticated Deception* clips were significantly longer than *Plain Truth* or *Simple Deception* clips (*p*s < 0.001), while *Plain Truth* and *Simple Deception* were not significantly different from each other (*p*s > 0.1). Overall, deceptive plays lasted longer than truthful plays in this game. Regarding to the two deception strategies, *Sophisticated Deception* last longer than *Simple Deception*, consistent with increased cognitive demands in the *Sophisticated Deception* condition. The Media Richness Theory was also reflected by longer overall duration in *Sophisticated Deception* in nonspeaking condition, compared with that in the speaking condition.

#### Direct analysis

For each of the 25 facial indicators, we analyzed the indicator value (for static images) and the maximum indicator value (for video clips). In each case, these values were submitted to a 3 × 3 repeated-measure ANOVA with the within-subjects factors of *Dynamism* (static, nonspeaking, speaking) and *Deception Strategy* (*Plain Truth*, *Simple Deception*, *Sophisticated Deception*). To focus on facial regions that have previously been implicated in deception, we defined as indicators of interest (IOIs) those related to gaze aversion (gaze_angle_x and gaze_angle_y), pupil dilation (AU7 Lid Tightener, diameter of each pupil, separation between eyelids), chin raising (AU17), lip pressing (AU23), and facial pleasantness. Cheek Raiser (AU6) and Lip Corner Puller (AU12) are the two reliable indicators predicted by Ekman and Friesen (1978, 1982) to signal happiness^33,34^.

##### Main effect of Deception Strategy

As can be seen from Table 3, thirteen indicators were significantly more intense in *Sophisticated Deception* than in *Plain Truth*. In contrast, only AU15 (Lip Corner Depressor) and AU17 (Chin Raiser) were more intense in *Simple Deception* than in *Plain Truth*. Consistent with our main hypothesis, more action units were engaged in *Sophisticated Deception* than in *Simple Deception*.

**Table 3:** Indicators showing the main effect of Deception Strategy.

Indicator	Plain Truth		Simple Deception		Sophisticated Deception		*F*	*p*	η_p_^2^
	*M*	*SE*	*M*	*SE*	*M*	*SE*			
Eyebrows									
AU1 Inner Brow Raiser	0.64	0.05	0.71	0.05	0.83	0.07	4.05	0.04	0.09
Eyes									
AU7 Lid Tightener	1.48	0.12	1.45	0.11	1.89	0.12	16.14	< 0.001	0.29
gaze_angle_x (left–right)	0.03	0.01	0.04	0.01	0.05	0.01	5.64	0.01	0.13
Separation between eyelids	29.76	1.14	29.93	1.09	27.38	1.24	6.09	0.01	0.14
Nose									
AU9 Nose Wrinkler	0.30	0.02	0.33	0.03	0.39	0.03	3.89	0.03	0.09
Mouth									
AU10 Upper Lip Raiser	0.48	0.08	0.54	0.11	0.95	0.10	22.28	< 0.001	0.36
AU12 Lip Corner Puller	0.74	0.10	0.69	0.10	1.30	0.13	20.88	< 0.001	0.35
AU15 Lip Corner Depressor	0.36	0.02	0.46	0.03	0.49	0.03	15.82	< 0.001	0.29
AU17 Chin Raiser	0.79	0.06	0.94	0.07	1.18	0.07	20.81	< 0.001	0.35
AU23 Lip Tightener	0.33	0.03	0.34	0.03	0.42	0.04	5.18	0.01	0.12
AU25 Lips Part	1.05	0.05	1.10	0.05	1.27	0.06	8.17	0.001	0.17
Cheek									
AU6 Cheek Raiser	0.65	0.08	0.59	0.09	1.15	0.10	24.50	< 0.001	0.39
AU14 Dimpler	0.81	0.09	0.89	0.10	1.35	0.12	14.84	< 0.001	0.28

*SE* referes to stardard error.

##### Main effect of Dynamism

As shown in Table 4, most indicators in the upper face were significantly more intense in the static condition (the leanest medium) compared with the dynamic conditions. However, most indicators in the lower face were significantly more intense in the speaking condition (the richest medium) compared with the static condition. In hindsight, our hypothesis that overall intensity would increase from rich media to lean media was too simplistic, as speech disproportionately engages the lower face. However, for the upper face, which is less strongly engaged in speech production, the intensity effect was in the predicted direction (static > dynamic).

**Table 4:** Indicators showing the main effect of Dynamism.

Indicator	Static		Dynamic nonspeaking		Dynamic speaking		*F*	*p*	η_p_^2^
	*M*	*SE*	*M*	*SE*	*M*	*SE*			
Eyebrows									
AU1 Inner Brow Raiser	0.93	0.10	0.58	0.05	0.66	0.06	5.94	0.01	0.13
AU2 Outer Brow Raiser	0.91	0.08	0.45	0.05	0.45	0.04	19.38	< 0.001	0.33
Ratio of left eyebrow	5.43	0.08	5.85	0.08	5.88	0.09	113.86	< 0.001	0.75
Eyes									
AU7 Lid Tightener	0.93	0.09	1.86	0.12	2.03	0.12	115.24	< 0.001	0.75
AU45 Blink	0.77	0.11	1.88	0.14	1.64	0.15	16.92	< 0.001	0.30
gaze_angle_y (up-down)	0.08	0.01	0.23	0.01	0.21	0.01	52.18	< 0.001	0.57
Diameter of left pupil	47.55	0.94	13.21	0.16	12.26	0.18	1614.91	< 0.001	0.98
Diameter of right pupil	48.15	0.72	13.54	0.19	13.48	0.21	3049.60	< 0.001	0.99
separation between eyelids	55.14	2.23	16.06	0.48	15.87	0.54	475.78	< 0.001	0.92
Nose									
AU9 Nose Wrinkler	0.53	0.06	0.23	0.01	0.26	0.01	21.47	< 0.001	0.36
Mouth									
AU10 Upper Lip Raiser	0.68	0.09	0.49	0.08	0.80	0.10	12.10	< 0.001	0.24
AU12 Lip Corner Puller	0.96	0.08	0.78	0.11	0.99	0.11	5.23	0.01	0.12
AU15 Lip Corner Depressor	0.32	0.06	0.41	0.02	0.58	0.03	13.46	< 0.001	0.26
AU17 Chin Raiser	0.86	0.09	0.82	0.05	1.23	0.09	10.33	< 0.001	0.21
AU20 Lip Stretcher	0.69	0.08	0.37	0.02	0.50	0.03	10.08	< 0.001	0.21
AU23 Lip Tightener	0.33	0.04	0.31	0.03	0.45	0.03	6.98	0.003	0.15
AU25 Lips Part	0.55	0.08	0.59	0.04	2.30	0.11	133.39	< 0.001	0.77
Ratio of mouth	0.33	0.01	0.35	0.01	0.53	0.01	318.16	< 0.001	0.89
Cheek									
AU6 Cheek Raiser	0.85	0.08	0.68	0.09	0.86	0.09	5.29	0.01	0.12
AU14 Dimpler	0.85	0.09	1.07	0.10	1.13	0.10	7.26	0.003	0.16
Jaw									
AU26 Jaw Drop	0.41	0.07	0.62	0.03	1.87	0.09	143.90	< 0.001	0.79

*SE* referes to stardard error.

##### Interactions between Deception Strategy and Dynamism

The interaction results identified different regions of facial activation for the different dynamism conditions. Several of these regions distinguish *Sophisticated Deception* from the *Simple Deception* and *Plain Truth* conditions. As shown in Table 5, the most diagnostic regions were in the lower face, with AU12 (Lip Corner Puller) and AU17 (Chin Raiser) showing significantly more activation in *Sophisticated Deception* relative to *Plain Truth* and *Simple Deception* in all three dynamism conditions. AU14 (Dimpler) activation was significantly more intense for *Sophisticated Deception* only in the dynamic conditions, whereas AU26 (Jaw Drop) was significantly more intense for *Sophisticated Deception* only in the nonspeaking condition. In the speaking condition, the ratio of mouth metric was significantly lower in *Sophisticated Deception* relative to *Plain Truth* and *Simple Deception*. In the static condition, only AU17 was significantly more intense in *Simple Deception* than in *Plain Truth*. There were no other significant differences between deception strategies. To summarize, the *Sophisticated Deception* strategy led to more intense indicators in the dynamism conditions, compared with *Simple Deception* and *Plain Truth* strategies.

**Table 5:** Indicators showing interaction between Dynamism condition and Deception Strategy.

Indicator	*F*	*p*	η_p_^2^
Mouth			
AU12 Lip Corner Puller	4.33	0.003	0.10
AU17 Chin Raiser	3.31	0.02	0.08
Ratio of mouth	3.08	0.03	0.07
Cheek			
AU14 Dimpler	5.26	0.001	0.12
Jaw			
AU26 Jaw Drop	4.31	0.002	0.10

##### Indicators of interest (IOI) analysis

**Figure 2:**
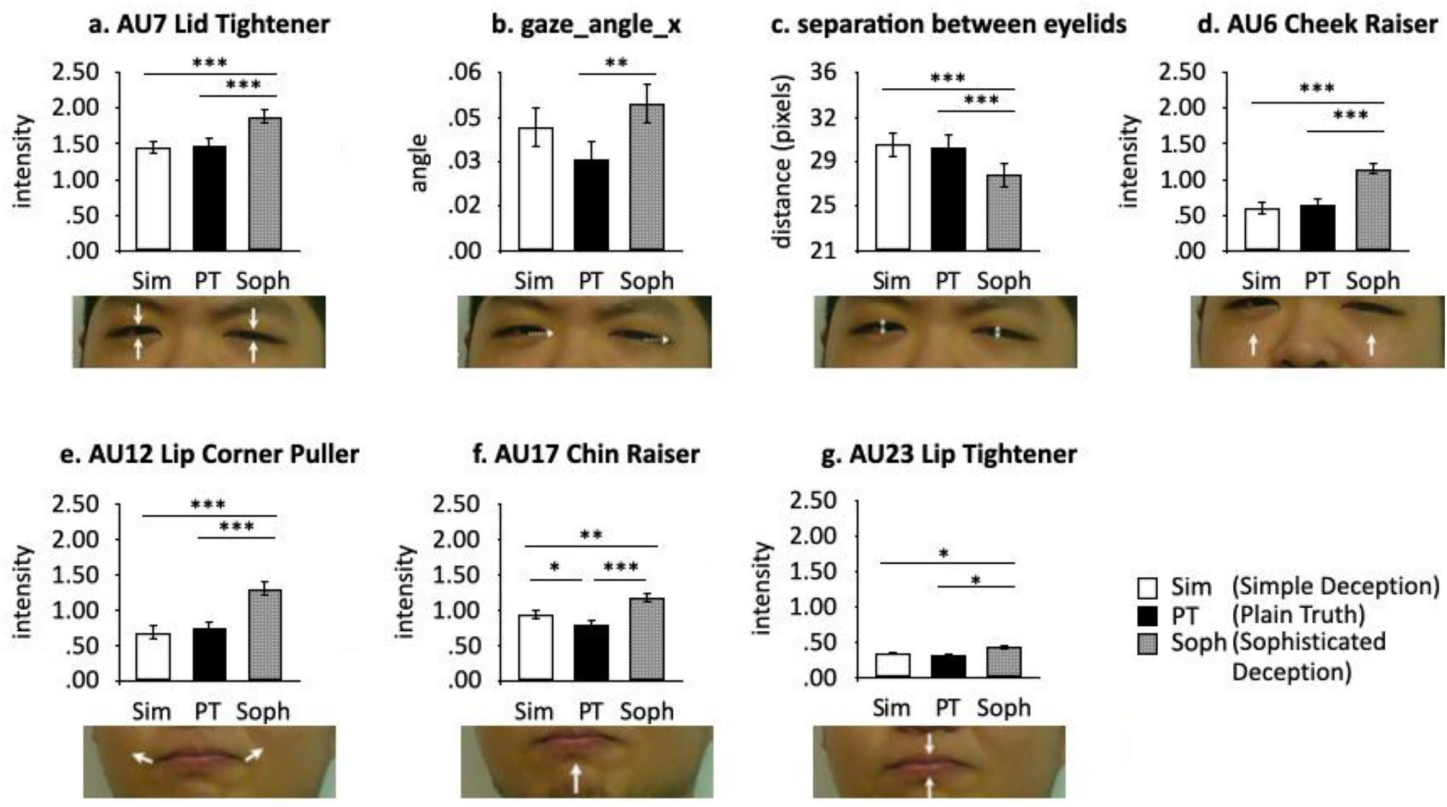
The main effect of Deception Strategy observed in IOIs. Each bar chart is followed by the corresponding face region from the screenshot of the participant’s face in the static condition, generated from the current research. Arrows show AU region and direction of motion. Dotted line arrows show gaze direction. Double-ended arrows show separation between eyelids. Error bars indicate standard error. ^*^p < .05; ^**^p < .01; ^***^p < .001.

**Figure 3:**
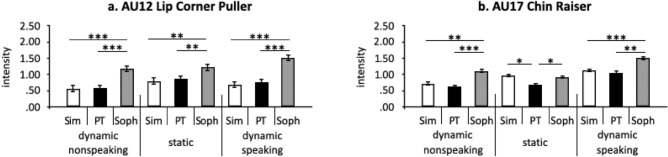
Summary of simple main effects of Deception Strategy for each IOI. Error bars indicate standard error. ^*^p < .05; ^**^p < .01; ^***^p < .001.

IOIs in gaze_angle_x, gaze_angle_y, AU7 (Lid Tightener), diameter of each pupil, separation between eyelids, AU17 (Chin raised), AU23 (Lip pressing), AU6 (Cheek Raiser), and AU12 (Lip Corner Puller) were defined according to previous studies^8,35,36^. Separate 3 (Deception Strategy) × 3 (Dynamism condition) ANOVAs on indicator values revealed significant main effects of Deception Strategy in AU6, AU7, AU12, AU17, AU23, gaze_angle_x, and separation between eyelids (see Fig. 2), as well as significant interactions in AU12 and AU17 (see Fig. 3).

The Indicators of Interest (IOIs) in this study were derived from previous studies on facial cues to *Simple Deception* and observers’ stereotypical beliefs about *Simple Deception*. Consistent with our main hypothesis, all of these IOIs were significantly more intense in *Sophisticated Deception* relative to *Simple Deception*, except for the diameter of both pupils—physiological indicators that might be hard for deceivers to control voluntarily. Among all the IOIs, only AU17 (Chin raiser) carried information that could be used for *Simple Deception* detection, and even this applied to the static condition only.

### Metacognitive analysis

#### Subjective AU intensity from the self-report retrospective questionnaire

To supplement the analysis of the 17 AUs which could be extracted from OpenFace 2.0, we also asked participants to rate their own facial behaviour in each deception strategy. Separate one-way ANOVAs revealed significant differences in estimated intensity for AU4, AU17, AU23 and AU61 (Eyes Turn Left) between the three strategies (*F*’s > 4.09, *p*’s < 0.02). Participants reported higher intensity of AU4, AU17 and AU61 for *Sophisticated Deception* than for *Plain Truth*, and higher intensity of AU23 in *Simple Deception* than for *Plain Truth*. As expected, participants underestimated the number of AUs engaged during their own *Sophisticated Deception*, compared with the objective measures of AU intensity by OpenFace 2.0. Participants also overestimated the intensity of their brow lowerer (AU4) in *Sophisticated Deception* and their lip tightener (AU23) intensity in *Simple Deception*.

#### Estimation of observers’ performance

For each deception strategy, participants were also asked to estimate the proportion of selectors who would choose the card the participant intended (0–100). One-sample t-tests showed that, for each strategy, participants estimated significantly more than 50% of selectors would choose the intended card (*Plain Truth* strategy [*M* = 64.05, *SE* = 2.14; *t* (39) = 6.57, *p* < 0.001, *d* = 1.04]; *Simple Deception* strategy [*M* = 58.05, *SE* = 2.08; *t* (39) = 3.88, *p* < 0.001, *d* = 0.61]; *Sophisticated Deception* strategy [*M* = 58.63, *SE* = 2.75; *t* (39) = 3.13, *p* < 0.01, *d* = 0.50]). There was no significant difference between the three strategies [*F* (2, 78) = 2.65, *p* = 0.08, η_p_^2^ = 0.06], indicating similar confidence of success across these conditions.

## Discussion

The current findings span cognitive and metacognitive levels. We begin by summarizing the findings at each level in the context of previous research. We then consider theoretical and applied implications of the findings as a whole.

### The cognitive level

#### Indirect indicators

Our analysis of clip durations showed that players spent longer on *Sophisticated Deception* than either *Simple Deception* or *Plain Truth*—especially when they were unable to use voice cues. This finding is consistent with Cognitive Load theory, in that lying requires more cognitive effort than telling the truth^37^. On this view, deception by telling the truth—*Sophisticated Deception*—requires particular mental effort. Relative to the demands of *Simple Deception*, *Sophisticated Deception* requires a further mental leap, as the player’s expectation (that selectors don’t trust them) conflicts with their speech (the truth). Alternatively, *Sophisticated Deceivers* might deliberately slow their expression to increase their chances of being suspected. Either way, the differences between conditions suggest that duration of facial behavior could be an indirect indicator of *Sophisticated Deception*.

#### Direct indicators

More direct indicators of deception have been sought in facial behavior. In the current study, we assessed differences in facial action units, pupil dilation, blink separation, and gaze direction, as well as ratio measures in the eyebrow region and the mouth region. Below we summarize possible cues to *Simple Deception* and *Sophisticated Deception* in these facial indicators.

(i) *Detecting Simple Deception. Simple Deception* and *Plain Truth* share the same expectation about the selector’s belief, which is that they are hearing the truth. Although deceivers in this study pretended that they were telling the truth, their facial behaviors could still leak their true intentions. Lip corner depressor (AU15), which is a reliable indicator of felt sadness^38^, and chin raiser (AU17), intensified in *Simple Deception* relative to *Plain Truth*. The latter finding is in line with the meta-analysis of DePaulo et al.^8^, in which lying was associated with increased chin raise and decreased facial pleasantness. We found no other facial cues to *Simple Deception*, consistent with the overall message from the literature that detecting deception from the face is difficult and unreliable.
(ii) *Detecting Sophisticated Deception.* We saw that *Simple Deception* and *Plain Truth* were difficult to distinguish based on AU activations. However, the situation for *Sophisticated Deception* was very different. The same quantitative analysis of facial AUs revealed several reliable indicators of *Sophisticated Deception*, supporting our main hypothesis. In *Sophisticated Deception*, players were telling the truth but were trying to make others believe that they were lying. How might players accomplish this goal? We suggest that their self-presentations will be informed by stereotypical beliefs about facial cues to deception (whether or not those beliefs are accurate). Having settled on a set of cues, players may deliberately leak those cues in a bid to convince others that they are lying. One way to leak cues deliberately would be to exaggerate the intensity of relevant AU activations. Consistent with this proposal, we found that stereotypical cues to deception were more intense in the *Sophisticated Deception* condition than in the *Plain Truth* condition. The same was true for deception IOIs, relating to visibility of the eyes, chin elevation, and facial pleasantness. In sum, facial behavior was more exaggerated in *Sophisticated Deception* than in *Simple Deception*. Moreover, this divergence increased when communication bandwidth was restricted. Comparisons of dynamic speaking, dynamic non-speaking, and static conditions show that deceivers pushed their facial behavior hardest in the static condition when they had fewest other cues to work with^17^.

Interestingly, we also saw more traces of basic facial expressions of emotion in *Sophisticated Deception*, specifically AU activations associated with sadness, disgust, and surprise. These observations recall an early proposal in deception research that exaggerated expressions and unusual combinations of AUs might be used to detect lies^10^. Support for this idea has been weak among studies that focused on *Simple Deception*^8,11^. However, exaggerated or unusual expressions may bolster *Sophisticated Deception* if they increase the selector’s suspicion.

It is important to reiterate that players’ stereotypical behaviors did not necessarily reflect accurate cues to deception. The example of gaze aversion (looking away from the conversation partner) is illustrative. Gaze aversion is widely believed to be a cue to deception^35^. However, the evidence is that gaze aversion is not in fact a reliable cue to deception (see DePaulo et al.^8^ for meta-analysis). What is striking about facial behaviors in the current study is that they reflect both of these elements—the stereotypical belief and the matter of fact. *Sophisticated Deception* and *Plain Truth* were significantly different in terms of gaze aversion, as players relied on shared stereotypical beliefs. In contrast, *Simple Deception* and *Plain Truth* were not significantly different, reflecting the finding that gaze aversion and lying are unrelated. Although we focus on gaze aversion to illustrate this point, the same pattern applies to other facial behaviors that are erroneously associated with lying. Generally, those behaviors emerged in the *Sophisticated Deception* condition, but not in the *Simple Deception* or *Plain Truth* conditions. The key to the *Sophisticated Deception* strategy is the common ground between deceivers and selectors. Provided that selectors share the deceiver’s stereotypical beliefs, they are liable to mistake *Sophisticated Deception* for *Simple Deception*, which conforms exactly to the deceiver’s wishes.

### The metacognitive level

There are two facets to the metacognitive findings—players’ estimation of their own facial behaviors and players’ estimation of selectors’ accuracy in detecting deception. Taking these in turn, players reported fewer AUs intensified in *Sophisticated Deception* compared with the objective results, but the self-report data give a clear indication that some AUs were intended. Evidently, players’ awareness of their own facial behaviors was limited. Machine vision could detect more subtle changes in the intensity of facial movements than the humans who were producing them.

As for players’ estimation of selectors’ performance, the main message is that players’ confidence was above chance in all conditions, and showed no significant difference across *Plain Truth*, *Simple Deception*, and *Sophisticated Deception* conditions. Interestingly, we didn’t see that players felt *less* confident during *Sophisticated Deception*, even though this was a more elaborate strategy. Stromwall et al.^39^ demonstrated that lay and professional individuals rely on apparent nervousness to detect deception. The metacognition results presented here suggest that when people deceived by telling the truth, they were no more nervous than if they were simply cheating or being honest. Future studies could test this possibility directly by measuring physiological markers such as skin temperature and heartbeat. They could also test whether players’ estimations were accurate by incorporating the perspective of selectors.

### Implications

The present findings deepen our understanding of deception cues in several ways. First and foremost, we extend the research of facial cues to deception by including *Sophisticated Deception*. The current work also enriches integration between cognition and computer vision, using machine learning to quantify psychological variables in a social task. Our findings demonstrate that the Joker Game is a useful paradigm for eliciting *Plain Truth*, *Simple Deception*, and *Sophisticated Deception* behaviors in a manner that combines naturalistic gameplay with experimental control. We identify at least three advantages to this approach: (i) Observers can make veracity judgments mainly based on facial behaviors. As a counterexample, the sender-receiver game in decision-making research on *Sophisticated Deception* relies on payoff amounts of both sides to drive judgments^4^. (ii) The rules of the game accommodate all three deception strategies (*Plain Truth*, *Simple Deception*, and *Sophisticated Deception*). As a counterexample, the *Rock-Scissors-Paper* game can accommodate the two deception conditions, but not the truth condition. (iii) The task allowed players to deceive across all three *Dynamism* conditions (static, dynamic nonspeaking, and dynamic speaking). Embedding sophisticated deception in the Joker game gives rise to an ‘Honest Joker’ phenomenon, in which facial expression is used to turn the truth against an opponent.

Our findings point to a number of directions for future research. First, individual differences in facial cues to deception remain relatively unexplored. We suggest that two aspects are worth distinguishing—inter-personal differences and intra-personal differences. Previous work has shown that facial cues to deception can be highly idiosyncratic^8^. For example, extroverted individuals tend to decrease their movement when lying, whereas introverted individuals make more frequent movements^40^. Thus, the influence of personality on facial expressions under different deception strategies seems a promising avenue. Moreover, the same person may exhibit different behaviors in different situations (intra-individual differences). For instance, cues to deception from facial expression and gaze behavior could evolve during an interaction as evidence of success or failure accumulates. Future variants of the Joker Game experiment could bring deceivers and selectors together in person to test whether deceivers’ immediate reactions differ in a face-to-face situation with eye contact. Furthermore, detection accuracy for sophisticated deception remains an open question. A meta-analysis found that detection rates for simple deception were close to chance levels, but the situation may be different for sophisticated deception, given its reliance on shared stereotypes^41^. Finally, we acknowledge that a more complete model of deception detection will have to combine cues from face, voice, and posture. We make available the Joker Game stimuli (120 photographs; 240 videos) as a contribution to this broader research effort. For now, we demonstrate the Honest Joker phenomenon and use it to probe stereotypical beliefs about facial cues to deception.

### Supplementary Information

Supplementary Information.

## Data Availability

The datasets and materials generated during and/or analysed during the current study can be accessed via the following link https://osf.io/t2bp6/?view_only=28355ed426be456d8771b1a454efbbf7
