# Supplementary material for: An Honest Joker reveals stereotypical beliefs about the face of deception
Source: Sci Rep. 2023 Oct 3;13:16649. doi: 10.1038/s41598-023-43716-4 (PMC10547800; doi:10.1038/s41598-023-43716-4)
Supplement: Supplementary file 1 — Supplementary Information. [file 41598_2023_43716_MOESM1_ESM.docx]

### Supplementary Information

**Verbatim task instruction**

*“A standard 52-card deck comprises 13 ranks in each of the four suits: clubs (♣), diamonds (♦), hearts (♥) and spades (♠). Each card can be paired according to its rank. If we add a single Joker to the deck, then this unmatchable Joker card becomes the ‘ghost card’. The dealer shuffles and deals all cards to the players, one card at a time. Players look at their cards and discard any pairs they have (e.g., two kings, two sevens, etc.) face up. Each player takes turns offering his/her hand face-down to the person on his/her left, keeping the face of all cards unexposed. That person selects a card and adds it to his hand. This player then checks if the selected card makes a pair with any of his/her original cards. If so, the pair will be discarded face up as well. The player who just took a card then offers his/her hand to the person on his left, and so on. The object of the game is to continue to keep picking up cards and discarding pairs until no more pairs is available. Whoever holds the ‘ghost card’ at the end of the game loses.*

*Interestingly, before deciding which card to choose, the player (receiver) have a chance to tentatively ask the player whose card is going to be selected (sender) whether the card he/she pretends to choose is safe for him/her, that is, the card is not the ghost card. The receiver doesn’t know the card’s face, because all cards are face-down, but the sender knows. So, the receiver will make the final decision based on the sender’s facial expressions.*

*Now,* *imagine you are the sender in the last few turns with only two cards in your hand. The other player is going to choose one card from your hand.* *Before deciding which one to choose, the player has a chance to ask you tentatively which one is safe for him/her and make the final decision based on your facial expressions. You may have either two safe cards or one safe card and one Joker card, which results in three kinds of responses.*

*The first one is to be honest when you have two safe cards, we call it Plain Truth. You don’t have to deceive the receiver, because no matter which card is picked, you’re always safe. Note, under this condition, we assume the receiver also has no Joker card before selecting your card. The Joker card might in another player’s hand. When you have one Joker card, no doubt you’re eager to see the receiver to take it away. So, you may have two deception strategies to increase the receiver’s chance of picking the ghost card. One is Simple Deception, the most common deception strategy by sending the wrong information but intending the receiver to believe it as a truth. That is, when the receiver touches the Joker card and ask if this card is safe for him/her, you should answer that it is a safe card and try to think about how to make him believe your answer with the help of your facial expressions, and vice versa for the actual safe card. The other form of deception is the Sophisticated Deception. Sometimes, whatever you say would be suspected. Using the Sophisticated Deception strategy, you may deliberately tell the receiver the truth but induce the receiver to perceive it as a lie through your facial expressions.”*

###
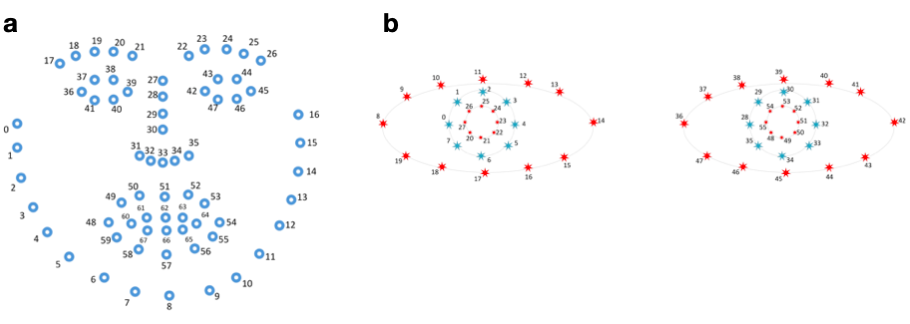


**Figure S1.** The landmark index of the whole face (a) and the eye region (b).

**Table S1.** Denotation of Euclidean Distance between facial landmarks

|  | Denotation | Distance between two landmarks |  | Denotation | Distance between two landmarks |
| --- | --- | --- | --- | --- | --- |
| Left face  (Figure 4A) | ld1 | 19-39 | Right face  (Figure 4A) | rd1 | 24-42 |
|  | ld2 | 39-51 |  | rd2 | 42-51 |
|  | ld3 | 21-39 |  | rd3 | 22-42 |
|  | lfd | 27-39 |  | rfd | 27-42 |
|  | md1 | 50-58 |  | md3 | 52-56 |
|  | md2 | 51-57 |  | md4 | 48-54 |
| Left eye  (Figure 4B) | Lpupil_d | 21-25 | Right eye  (Figure 4B) | Rpupil_d | 49-53 |
|  | Blink_left | 11-17 |  | Blink_right | 39-45 |
